# Perinatal mental health around the world: priorities for research and service development in Sweden

**DOI:** 10.1192/bji.2019.23

**Published:** 2020-02

**Authors:** Birgitta Wickberg, Marie Bendix, Margareta Blomdahl Wetterholm, Alkistis Skalkidou

**Affiliations:** 1 Associate Professor, Department of Psychology, University of Gothenburg, Göteborg, Sweden. Email: birgitta.wickberg@gmail.com; 2 Liaison Perinatal Consultant Psychiatrist, Centre for Psychiatry Research & Stockholm Health Care Services, Stockholm County Council/Karolinska Institutet Stockholm, Sweden; 3 Professor, Department of Women's and Children's Health, Uppsala University, Sweden

**Keywords:** Universal screening, multidisciplinary teams, immigrants

## Abstract

Sweden has a unique opportunity to identify and follow up women presenting with, or at risk for, perinatal mental health problems and disorders because universal screening programmes are provided by its primary healthcare system. Although they are implemented across almost the entire population, screening programmes are not necessarily leading to effective interventions because the multidisciplinary perinatal mental healthcare teams that provide for the assessment and treatment of moderate to severe disorders are very few in number and must be increased. In particular, efforts to reach immigrant parents must be intensified to achieve equal quality of care for all.

Sweden, a Nordic country with ten million inhabitants, has in one generation changed from an ethnically homogeneous country to one of the most heterogeneous in Europe. Twenty-four per cent of the population is of foreign origin, either born abroad or having both parents born abroad.^1^ Whatever their reason for migrating to Sweden, all immigrant groups report having poorer health, including mental health, than the rest of the population.^2^ Over the past decade, mental health problems have increased, and psychiatric diagnoses are now the most common reason for sick leave, the proportion having more than doubled between 2010 and 2015. Adjustment disorders and other reactions to severe stress account for almost two-thirds of the total increase in sick leave, especially among people in younger middle age.^3^ There are many possible factors contributing to this increase, such as the stress inherent in trying to balance the demands of work and family life. One way to identify mental health needs in the population as a whole is to focus on the childbearing period. At this time, people are usually open to lifestyle changes and to receiving care, while they are in very close contact with the healthcare system. This offers an optimal opportunity for primary prevention and early interventions.

## Antenatal and child healthcare services

In Sweden, the antenatal and Child Health Services (CHS) have a long tradition, are free of charge and almost 100% of pregnant women and new parents with infants attend regular appointments. Antenatal care is mainly delivered by midwives and offers 8–11 recommended check-ups during pregnancy, and one postpartum appointment. The child healthcare programme is offered to all children between 0 and 5 years as well as parents, regardless of income or risk exposure. In recent decades there has been a shift from what used to be primarily somatic management concerns about pregnancy and postnatal care towards an increasingly public health and psychosocial perspective. The CHS team is led by a nurse, and includes a general practitioner or a paediatrician and, in almost all counties of Sweden, a psychologist. These psychologists work within the antenatal and child healthcare systems, offering regular consultation meetings with midwives and nurses and providing easy access for assessment and treatment to women with perinatal mental health problems.

## Early identification

Much research in perinatal mental health has focused on postnatal depression, but in the past few years this perspective has broadened to include mental illness during pregnancy and a wide range of other mental disorders. Programmes that combine the systematic identification of postnatal depression with enhanced support have proven to be both clinically valuable and cost-effective (for a review see Howard *et al*^4^). Whether universal prenatal screening for depression is clinically effective and cost-effective is still under discussion. Some counties in Sweden are nevertheless implementing pilot projects. Antenatal screening for depression and other current or past mental health problems is by questionnaires or structured interviews.^5^ One county recently introduced a telephone call, 2 weeks after delivery, to ask about the mother's mental state. The effectiveness of these approaches in improving postnatal health is not yet established.

In 2010, the Swedish National Board of Health and Welfare recommended postnatal screening for depression using the Edinburgh Postnatal Depression Scale (EPDS), to be followed by person-centred counselling (so-called ‘listening visits’) by CHS nurses for all women with subthreshold or minor depression. This procedure is now implemented all over the country.^6^ National figures on postnatal screening rates are not available but in the three largest regions of Sweden, which include more than half of the population, about four out of five women took part in the screening visit 6–8 weeks postpartum during 2017. In the southern region, 87% of all new mothers were screened and 10% were followed up, half of them with ‘listening visits’ and the other half with a referral to psychologists or general practitioners.^7^ Despite these achievements, it has proved challenging to reach immigrant mothers, who are less likely to be offered counselling and less often agree to participate in screening; this group is at especially high risk of postnatal depression.^8^

## Perinatal psychiatry

Women at risk of, or suffering from, severe mental disorders require rapid access to perinatal care from specialist psychiatric units. There is a need for multidisciplinary cooperation between psychiatry and antenatal/CHS and social services, but Sweden lacks specialised perinatal psychiatric services. Ideally, the management of perinatal mental disorders is provided by experts with appropriate knowledge. For example, psychiatric units specialising in affective disorders may be able to provide prophylactic lithium treatment to women with severe affective spectrum disorders, but this necessitates collaboration with both obstetric and paediatric services.^9^

Access to treatment and care for women with severe mental disorders still needs considerable improvement. A study on maternal suicides in Sweden found that 47% of the 103 women who died by suicide between 1980 and 2007 had had psychiatric disorders before their pregnancy (79% had bipolar disorder or psychosis, substance use disorders or a history of suicide attempts). But fewer than half the women's antenatal and obstetric records had taken note of that psychiatric history and in only 18% was there any record of prior suicide attempts. Although 30% had had at least one consultation for mental health problems within the antenatal care system, only 8% had received in- or out-patient psychiatric care.^10^

To increase access to psychiatric care during pregnancy, some obstetric and psychiatric clinics have initiated consultation-based cooperative activities. In one of these services, about 5% of pregnant women had been referred to a perinatal psychiatric liaison team during the past 10 years. Referrals originated mainly from antenatal and primary healthcare centres, but also from obstetric and specialist psychiatric services.^11^ A perinatal liaison team can both offer treatment and act as a gateway linking the different healthcare providers needed to manage pregnant women with severe mental disorders.

Providing equal access to healthcare for all the population in Sweden is challenging because of the low population density outside our few larger cities. During recent years, there have been efforts to improve local knowledge about perinatal psychiatric disorders through the publication of regional and national guidelines and multidisciplinary educational projects. Providing increased collaboration between psychiatric and primary healthcare providers, social services and obstetric services, such projects will be more rapidly implemented than the development of specialised perinatal psychiatric services. Mother and baby units do not yet exist in Sweden, but their formation is under discussion.

## Priorities for research and service development

Our first priority is to ensure that there is rigorous assessment of the different screening protocols for risks to antenatal mental health. There should be national guidelines, based on scientific evidence, to complement the existing guidelines for screening in the postnatal period. Interventions for the prevention of postpartum depression tend to be more effective when targeting women at risk and also when offered early after delivery.^12^ Current projects are using machine learning and deep learning techniques to develop algorithms that will allow us to identify women at high risk of postpartum depression with greater accuracy. Further research efforts should be encouraged.^13^

Better organisation of perinatal mental healthcare services would improve collaboration between obstetric and psychiatric services, leading to more effective prophylaxis and treatment.^9^ There is a need to lower maternal suicide rates in Sweden; we should increase detection of those at risk, provide individualised planning and quick access to psychiatric care.^10^ Research priorities include discovering who is missing from existing screening programmes and we should plan interventions to address that problem.

## Conclusions

A universal screening programme for postnatal depression is implemented within the Swedish healthcare system but it does not necessarily lead to effective therapeutic action. A structured programme to detect and treat mental health problems during antenatal care, equivalent to perinatal mental health multidisciplinary teams, needs to be developed and evaluated in all parts of the country.
